# Plasma Galectin-3 is associated with progression from paroxysmal to persistent atrial fibrillation

**DOI:** 10.1186/s12872-021-02043-0

**Published:** 2021-05-02

**Authors:** Qianhui Wang, Li Xu, Ying Dong, Yuan Fu, Yuxia Pan, Qianran Luan, Ye Liu, Zheng Liu, Xinchun Yang, Mulei Chen, Yuanfeng Gao

**Affiliations:** Heart Center and Beijing Key Laboratory of Hypertension, Beijing Chaoyang Hospital, Capital Medical University, Beijing, 100020 China

**Keywords:** Atrial fibrillation; Biomarker; Progression; Galectin-3

## Abstract

**Background:**

Galectin-3 (Gal-3) is currently recognized as a promising biomarker for myocardial fibrosis. This study aimed to explore the potential association between plasma Gal-3 concentrations and atrial fibrillation (AF) progression in paroxysmal AF (PAF) patients

**Methods:**

A total of 213 PAF patients were included for analysis in this study. All peripheral blood samples were prospectively collected and stored at -80℃ for subsequent Gal-3 quantification. The AF progression was defined as transformation from PAF to persistent AF (PsAF).

**Results:**

A total of 51 PAF patients progressed to PsAF during a mean follow-up period of 674.44 ± 19.48 days. Patients with AF progression had significantly higher baseline plasma Gal-3 concentrations than those stayed in PAF status (13.52 ± 0.94 vs. 7.93 ± 0.37, *p* < 0.001). All PAF patients were divided into two subgroups based on the median value of plasma Gal-3 concentrations. Kaplan–Meier curve analysis showed a significantly higher AF progression rate in the higher plasma Gal-3 concentration group (log-rank test *p* < 0.001). In the Cox regression analysis, plasma Gal-3 concentration and left atrial diameter (LAD) were showed significantly associated with AF progression, even after adjustment of other potential confounding risk factors. Discrimination for AF progression with a simple model which consists of plasma Gal-3 concentration and LAD was modest with a C-statistic 0.72 (95%CI 0.64–0.80). Plasma Gal-3 concentration significantly improved the predictability by appropriately reclassifying several patients with progression (NRI = 28.3%, *p* = 0.003).

**Conclusion:**

Elevated plasma Gal-3 concentration is significantly associated with AF progression from PAF to PsAF. Plasma Gal-3 concentration could be used for PAF progression risk stratification and guiding management for PAF patients.

## Background

Atrial fibrillation (AF) is the most common sustained tachycardia arrhythmia in clinical practice [1]. AF patients often experience decreased quality of life and significantly greater risk of cardiovascular events including heart failure (HF), myocardial infarction (MI) [2], and ischemic stroke. AF prevalence increases with age and is estimated to double in the next 30 years [3, 4].

AF is a progressive disease and the majority of cases start with short, self-terminating paroxysmal AF (PAF), which gradually evolves to a longer persistent AF (PsAF) or even permanent AF status [5, 6]. AF progression is reported to be significantly associated with increased mortality and adverse cardiovascular outcomes [7]. Previous studies observed that catheter ablation was an effective and feasible approach in preventing AF progression, particularly for cases with PAF. However, a certain proportion of PAF patients progress to persistent or permanent type of AF even after catheter ablation, while some patients who did not undergo catheter ablation therapy remain in paroxysmal status for decades [8]. These observations indicated that the mechanisms underlying AF progression are complicated and a better understanding of risk factors associated with AF progression can help to guide more comprehensive management to prevent AF progression.

Inflammation and cardiac fibrosis have been proposed as the main contributors for AF on-set and sustenance [9, 10]. Galectin-3 (Gal-3), a member of the lectins family, which bind to β-galactoside, has been reported to be involved in regulating many conditions, including HF [11], hepatic and pulmonary fibrosis [12]. Recent studies revealed that elevated plasma Gal-3 concentrations were positively correlated with AF onset and recurrence after catheter ablation [13, 14]. However, data evaluating the value of plasma Gal-3 concentration in predicting progression for PAF patients is limited. Thus, in this study, we aimed to investigate the association between baseline plasma Gal-3 concentrations and AF progression in PAF patients.

## Methods

### Study subjects

A total of 213 consecutive symptomatic non-valvular PAF patients admitted to our institution between June 2016 and November 2018 were screened for this prospective study. Patients who met any of the following conditions were excluded from this study: malignant tumors, acute inflammatory diseases or chronic autoimmune diseases, AF secondary to hyperthyroidism, left ventricular ejection fraction (LVEF) < 30%, recent myocardial infarction (30 days), stroke history (< 6 months), and recent cardiac surgery (< 1 month). All baseline data, including clinical characteristics, demographic data, laboratory data, echocardiogram, electrocardiogram, and electrophysiological (EP) characteristics were collected from the electronic medical record system of the hospital. Written informed consents were obtained from all participants, and this study was approved by the ethics review board of our institution.

### Gal-3 measurement

Peripheral blood samples from each consenting patient were drawn into EDTA tubes at admission after overnight fasting. All samples were processed with centrifugation at 1000 g for 10 min within 4 h and stored as aliquots at − 80 °C for subsequent analysis.

Plasma Gal-3 concentrations were measured using the Immunoway (USA) KE1712, Human GAL-3 ELISA kit according to the kit manufacturer’s instructions.

### Follow-up approaches

Scheduled clinical visits and 12-lead ECG or 24 h Holter monitor were arranged at 1, 3, 6 months and every 3 months thereafter for all patients in the outpatient department. All patients were followed-up for at least 1 year. In addition to regular follow-up, unplanned visits or symptom-triggered self-reported AF episodes were also recorded. All patients were taught to judge the AF episodes by sensing the rate and strength of the radial artery. ECG and Holter results were recorded by telephonic follow-up if the patients could not visit our outpatient department. Patients were asked to report any documented AF recurrent episodes or suspicious symptoms of AF between scheduled visits.

In the present study, the main clinical end-point was progression from PAF to PsAF. The classification of AF in the present study was in accordance with the latest clinical guidelines for AF patients who underwent catheter ablation therapy [15]. AF episodes that self-terminated in < 1 week or terminated by any cardioversion measures, including antiarrhythmic drugs (AAD) and direct current cardioversion (DCC) were defined as PAF. Recurrent AF episodes lasting ≥ 7 days were defined as PsAF.

### Statistical analysis

All continuous variables were tested by Shapiro–Wilk test for the distribution of normality, and the results were listed as mean ± standard deviation (SD) or median and interquartile range. Student’s t-test or Wilcoxon rank-sum test was used to assess the intergroup differences, when necessary. Categorical variables were presented as numbers and percentages (%), and were tested by Chi-square test between groups.

All patients were divided into two subgroups by median value of Gal-3 concentration. Kaplan–Meier curve analysis was performed to calculate cumulative probability of freedom from AF progression and difference between the two groups was tested by log-rank test. Cox proportional hazards model regression analysis was used and a risk model was established to evaluate the potential risk factors for AF progression, and the results were recorded as hazard ratios (HR) and 95%CI. The value of discrimination for AF progression was assessed and showed with a C-statistic. We also calculated the net reclassification improvement (NRI) to evaluate the improvement of predictive ability of Gal-3 for AF progression compared with traditional risk factors. A two-tailed *p* value < 0.05 was considered to be statistically significant. All statistical analyses were tested by SPSS version 24.0, except the C-statistic and NRI. The C-statistic and NRI were calculated and tested by R version 4.0.3.

## Results

### Baseline characteristics of the subjects

AF progression events occurred in 51 of the 213 PAF subjects included in the study. The baseline characteristic comparison between patients with and without AF progression is as shown in Table 1. Patients with AF progression were older and had significantly higher BNP (1488.6 ± 339.19 vs. 551.1 ± 81.85, *p* < 0.001) and Gal-3 (13.5 ± 0.94 vs. 7.9 ± 0.37, *p* < 0.001) levels, larger left atrial diameter (LAD) (41.3 ± 0.90 vs. 39.2 ± 0.39, *p* = 0.021), and higher percentage of previous stroke history (17.7% vs. 7.4%, *p* = 0.035). Other clinical characteristics including gender, catheter ablation, hypertension, diabetes mellitus, high-sensitivity C reactive protein (hs-CRP), estimated glomerular filtration rate (eGFR), and body mass index (BMI) were comparable between the groups (*p* > 0.05).

**Table 1:** Baseline characteristics analysis

Variables	Progressed (51)	Not-progressed (162)	*p* value
Age	71.7 ± 1.7	66.9 ± 0.8	0.005
BMI, kg/m^2^	24.9 ± 0.5	25.4 ± 0.3	0.456
Male	33 (64.7%)	84 (51.5%)	0.068
Hypertension	36 (70.6%)	114 (69.9%)	0.539
Diabetes mellitus	16 (31.4%)	48 (29.5%)	0.461
Stroke	9 (17.7%)	12 (7.4%)	0.035
COPD	2 (3.9%)	1 (0.6%)	0.081
CAD	23 (45.1%)	56 (34.6%)	0.175
eGFR,mL/(min·1.73m2)	85.5 ± 4.58	86.1 ± 1.26	0.854
LAD,mm	41.3 ± 0.90	39.3 ± 0.39	0.021
LVEF,%	64.6 ± 1.19	64.6 ± 0.71	0.984
Gal-3,ng/ml	13.5 ± 0.94	7.9 ± 0.37	< 0.001
NT-proBNP,pg/ml	1488.6 ± 339.19	551.1 ± 81.85	< 0.001
hs-CRP,mg/ml	1.6 (0.92,4.70)	1.5 (0.91,3.36)	0.347
ACEI/ARB	23 (45.1%)	71 (43.6%)	0.486
Stains	32 (62.8%)	114 (69.9%)	0.213
β‐blocker	26 (51.0%)	81 (49.7%)	0.500
rhythm control	36 (70.6%)	134 (82.7%)	0.072
Ablation	33 (64.7%)	126 (77.8%)	0.061
AAD	3 (5.9%)	8 (4.9%)	0.790

BMI: Body mass index; COPD: chronic obstructive pulmonary disease; eGFR: estimated glomerular filtration rate; LAD: left atrial diameter; LVEF: left ventricular eject fraction; Gal-3: galectin-3;NT‐proBNP: N‐terminal pronatriuretic peptide; hs-CRP: high sensitive C‐reactive protein; ACEI: angiotensin converting enzyme inhibitor; ARB: angiotensin receptor blocker; AAD: anti-arrhythmic drugs

In the present study, 159 (74.6%) and 11 (5.2%) subjects reverted to sinus rhythm by catheter ablation and anti-arrhythmic drugs (amiodarone or propafenone), respectively, and rate control therapy was employed in the remaining 43 subjects due to contradictions or patient selection. There were no significant differences between patients that progressed to PsAF and those who did not, based on rhythm control strategies as well as rate control strategy (*p* < 0.05).

### Plasma Gal-3 elevation could be an independent risk factor for PAF progression

**Fig. 1:**
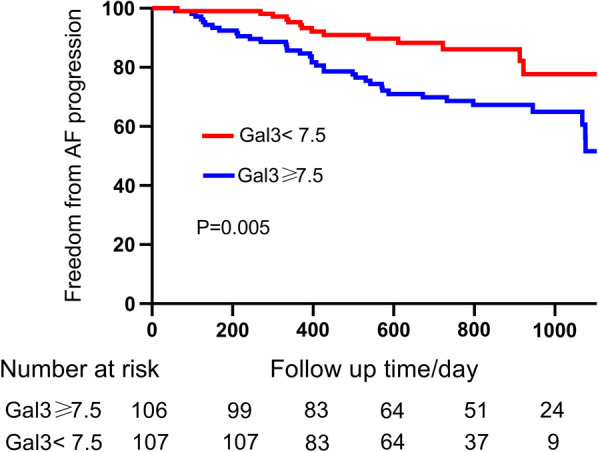
Kaplan‐Meier curve analysis shows the freedom from PAF progression for patients with Gal-3 ≥ 7.5 ng/ml and Gal-3 < 7.5 ng/ml. The blue line represents Gal-3 greater than or equal to 7.5 ng/ml and the red line represents Gal-3 less than 7.5 ng/ml

In the present study, we divided all patients into two subgroups based on the median value of Gal-3: high Gal-3 group (≥ 7.5 ng/ml) and low Gal-3 group (< 7.5 ng/ml). Kaplan–Meier curve analysis was conducted to evaluate the difference in PAF progression rates between the two groups. As shown in Fig. 1, it turned out that PAF patients with higher plasma Gal-3 concentrations had a significantly higher rate of AF progression (log-rank test, *p* < 0.001).

### Plasma Gal-3 was associated with progression from PAF to PsAF

The Cox proportional hazard model analysis was used for multivariate analysis to evaluate the simultaneous influence of prognostic factors on the progression of PAF (Table 2). In the univariate Cox regression analysis, in addition to Gal-3 (HR = 1.10, 95% CI 1.06–1.15, *p* < 0.001), other traditional known risk factors such as age (HR = 1.04, 95% CI 1.01–1.07, *p* = 0.010) and, LAD (HR = 1.07, 95% CI 1.01–1.12, *p* = 0.012) were also significantly associated with PAF progression. In the multivariate Cox regression analysis model, after adjustment of confounding factors, plasma Gal-3 concentration (1.10, 95% CI 1.05–1.15, *p* < 0.001) and LAD (1.06, 95%CI 1.02–1.11, *p* = 0.008) were remained independent risk factors for AF progression from PAF to PsAF. Discrimination for AF progression from PAF to PsAF with the simple risk model including Gal-3 and LAD showed a modest value with a C-statistic 0.72 (95%CI 0.64,0,80).

**Table 2:** Cox regression analysis

Variables	Univariable		Multivariable	
	HR (95% CI)	*p* value	HR (95% CI)	*p* value
Gal-3	1.10 (1.06–1.15)	< 0.001	1.10 (1.05–1.15)	< 0.001
Age	1.04 (1.01–1.07)	0.010	1.02 (0.99–1.05)	0.150
LAD	1.07 (1.01–1.12)	0.012	1.06 (1.02–1.11)	0.008
Stroke	1.31 (0.63–2.69)	0.469		
NT-proBNP	1.00 (1.00–1.00)	0.001		
hs-CRP	1.04(0.97–1.12)	0.283		

Gal-3: galectin-3; LAD: left atrial diameter; NT‐proBNP: N‐terminal pronatriuretic peptide; hs-CRP: high sensitive C‐reactive protein

We also calculated the NRI to evaluated the improvement of predictability of Gal-3 for AF progression. We first constructed a predictive model with age, LAD, and ischemic stroke history from the Cox regression analysis. Adding plasma Gal-3 concentrations to the predictive model showed a significant improvement in the predictability of AF progression by appropriately reclassifying several patients with AF progression (NRI = 28.3%, *p* = 0.003).

## Discussion

In the present study, we investigated the potential association between plasma Gal-3 concentration and progression from PAF to PsAF. The main findings were as follows: (1) plasma Gal-3 concentrations were significantly elevated in PAF patients with progression than those without; (2) plasma Gal-3 concentration was significantly associated with PAF progression, even after adjustment of other potential confounding risk factors; (3) the overall AF progression rate was 23.94% within a three-years follow-up period, and the majority of PAF progression events (42/51,82.35%) occurred during the first two-years of follow-up.

Increasing evidences have proposed cardiac fibrosis as the most prominent contributor in cardiac remodeling and perpetuating of AF [16]. Swartz, MF et al. reported elevated serum fibrosis markers and a higher percentage of cardiac fibrosis in patients who developed post-surgery AF than those without [17]. Okumura, Y et al. found that serum metalloproteinase (MMP)-2, one of the members of the MMP family, which are associated with wound healing and tissue remodeling, is significantly elevated in patients with AF recurrence after catheter ablation [17]. However, the specific mechanisms of cardiac interstitial fibrosis remain under-investigated. Recent studies have proposed that cardiac inflammation [18] and oxidative stress [19] might be key contributors to the development of the fibrosis pathway in AF patients.

Gal-3, a member of the beta-galactoside-binding proteins family, is secreted by activated macrophages and has been reported to be significantly up-regulated in several fibrotic and inflammation conditions [20, 21], including cardiac fibrosis [22]. Lili Yu et al. [23] demonstrated that cardiac fibrosis and HF progression were significantly attenuated in rats with genetic dysfunction or pharmacological inhibition of Gal-3. Umesh C Sharma et al. [24] conducted an animal study and found that Gal-3 was highly expressed in rats that subsequently developed HF than those that did not. Recombinant Gal-3 significantly promoted the proliferation and differentiation of myocardial fibroblasts and the deposition of collagen fibrous tissue. In addition, Yun-He Liu et al. [25] observed that continuous intrapericardial infusion of Gal-3 to rats significantly increased the expression of transforming growth factor-β (TGF-β) and Smad-3 phosphorylation, and induced cardiac fibrosis and hypertrophy. Moreover, these effects were mitigated by infusion with N-acetyl-seryl-aspartyl-lysyl-proline (Ac-SDKP), a natural tetrapeptide that prevents and even reverses cardiac inflammation and collagen deposition in patients with hypertension and HF. These results indicated that Gal-3 is also functionally associated with cardiac fibrotic activity.

Inflammation and subsequent cardiac remodeling have been reported to be significantly associated with AF pathophysiology [26]. Recent studies have suggested that increased Gal-3 concentrations are also involved in perpetuating AF [14, 27]. Gurses et al. performed a case–control study and revealed that serum Gal-3 concentrations were significantly different between AF patients and non-AF control patients: (1) serum Gal-3 concentrations were significantly higher in AF patients than non-AF controls; (2) PsAF patients also had significantly higher serum Gal-3 than PAF patients [28]. Diana Hernández-Romero1 et al. [29] found that patients with post-surgery AF had significantly higher serum Gal-3 concentration and severe cardiac fibrosis than those who had sinus rhythm. In addition, they also suggested that higher Gal-3 was an independent predictor for cardiac interstitial fibrosis. A recent meta-analysis proposed that elevated plasma Gal-3 levels were significantly correlated with recurrence of AF after catheter ablation [30]. Yoshio Takemoto et al. [31] demonstrated that patients with persistent AF had significantly higher intracardiac serum concentration of Gal-3, and Gal-3 was an independent predictor for AF recurrence after catheter ablation therapy. Using an animal model of PsAF, they further demonstrated that Gal-3 inhibition significantly mitigated cardiac remodeling by inhibiting TGF-beta signaling and reducing cardiac fibroblast activation. Hence, they proposed that Gal-3 inhibition therapy may decrease the overall AF burden, increase the possibility of AF termination (self-termination or termination by AAD drugs or DCC) and prevent AF progression.

In this study, the overall rate of progression was 23.94%, which was relatively higher than previous reports [6, 32]. Compared to these reports, our cohort had longer duration of follow-up and the patients were older, which may result in a higher progression rate. For example, Kerr C R et al. [6] reported that the rate of progression from PAF to PsAF at five years was 24.3%, while the patients were younger than our cohort (61.2 ± 14.2 vs. 68.12 ± 10.6). Cees B de Vos et al. [32] reported that progression from PAF to PsAF occurred in 15% of patients, but the patients were only followed-up for one year, which may have led to a lower rate of progression in their study. In the present study, we observed a significant difference in plasma Gal-3 concentration in patients with AF progression and identified that plasma Gal-3 was significantly associated with AF progression, even after adjustment of other confounding risk factors. As one of the most recognized factors in clinical practice to reflect the individual inflammation status, we also tested the association between hs-CRP and PAF progression, but no significant difference was found in the present study. In addition, we also found no significant correlation between Gal-3 and hs-CRP, suggesting that Gal-3 might regulate inflammation response via other pathways independent of hs-CRP.

A recent study conducted by De. With et al. [33] which investigated the factors associated with AF progression indicated that circulating Gal-3 concentration was not significantly associated with AF progression. However, this disparity between their study and ours could be due to the different selection of study population, follow up time and definition of AF progression (for example, change from persistent to permanent AF was also defined as AF progression in the study). Besides, in a recent meta-analysis performed by Blum et al. [34], which evaluated the incidence and factors associated with AF progression, also found that different population backgrounds like Age, hypertension, baseline AF type and follow-up duration could result in heterogeneity between studies. For example, PsAF patients at baseline had significant higher AF progression probability than those PAF patients (18.6% vs 7.1%). This could also in part underlie the disparity between our study and the study of De. With et al. Further studies or meta-analysis could be in need to further address the disparity.

## Limitations

This study had several limitations. First, plasma Gal-3 concentrations might be influenced by other fibrotic and inflammation conditions. However, in this study, we used strict selection criteria to exclude conditions that may influence the plasma Gal-3 levels, especially inflammation conditions like autoimmune diseases and acute cardiovascular diseases. Second, although a significant association between plasma Gal-3 concentration and AF progression was observed, we could not establish a cause–effect relationship. Further studies are needed. We did not obtain direct evidence of cardiac fibrosis like cardiac imaging data, but previous studies have demonstrated that plasma Gal-3 concentrations were significant positively associated with cardiac fibrosis degree. In the present study, majority of patients have undergone pulmonary vein isolation therapy, the cohort may be less representative of the whole PAF patients. While catheter ablation has been proved effective in restoring and maintaining sinus rhythm, more and more PAF patients would take catheter ablation therapy as first-line strategy. We did not offer continuous rhythm monitoring (7 days) for patients during follow-up, but we did have render multiple ECGs or Holters for patients suspicious of AF progression. Finally, the sample size with PAF progression was relatively small to generate conclusive opinion, but was valuable for hypothesis generation.

## Conclusion

Plasma Gal-3 concentration is significantly associated with AF progression from PAF to PsAF and might be used to help in risk assessment for PAF patients who are at greater risk of AF progression.

## Data Availability

The datasets generated and/or analyzed during the current study are not publicly available due to the restrictions of human genetics data policy of Beijing Chaoyang Hospital Ethics Committee, but are available from the corresponding author on reasonable request.

## Abbreviations

PAF: Paroxysmal atrial fibrillation
PsAF: Persistent atrial fibrillation
HF: Heart failure
MI: Myocardial infarction
Gal-3: Galectin-3
EP: Electrophysiological
LVEF: Left ventricular ejection fraction
AAD: Antiarrhythmic drugs, DCC:direct current cardioversion
hs-CRP: High-sensitivity C reactive protein
eGFR: Estimated glomerular filtration rate
BMI: Body mass index
LAD: Left atrial diameter
